# Development of an ELISA assay for the quantification of soluble huntingtin in human blood cells

**DOI:** 10.1186/1471-2091-14-34

**Published:** 2013-11-25

**Authors:** Luisa Massai, Lara Petricca, Letizia Magnoni, Luca Rovetini, Salman Haider, Ralph Andre, Sarah J Tabrizi, Sigurd D Süssmuth, Bernhard G Landwehrmeyer, Andrea Caricasole, Giuseppe Pollio, Simonetta Bernocco

**Affiliations:** 1 Pharmacology Department, Siena Biotech SpA, Strada del Petriccio e Belriguardo, 35, 53100 Siena, Italy; 2 Department of Neurodegenerative Disease, UCL Institute of Neurology, Queen Square, London WC1N 3BG, UK; 3 Department of Neurology, Ulm University, Oberer Eselsberg 45/1, 89081 Ulm, Germany

## Abstract

**Background:**

Huntington’s disease (HD) is a monogenic disorder caused by an aberrant expansion of CAG repeats in the huntingtin gene (*HTT*). Pathogenesis is associated with expression of the mutant (mHTT) protein in the CNS, with its levels most likely related to disease progression and symptom severity. Since non-invasive methods to quantify HTT in the CNS do not exist, measuring amount of soluble HTT in peripheral cells represents an important step in development of disease-modifying interventions in HD.

**Results:**

An ELISA assay using commercially available antibodies was developed to quantify HTT levels in complex matrices like mammalian cell cultures lysates and human samples. The immunoassay was optimized using a recombinant full-length HTT protein, and validated both on wild-type and mutant HTT species. The ability of the assay to detect significant variations of soluble HTT levels was evaluated using an HSP90 inhibitor that is known to enhance HTT degradation. Once optimized, the bioassay was applied to peripheral blood mononuclear cells (PBMCs) from HD patients, demonstrating good potential in tracking the disease course.

**Conclusions:**

The method described here represents a validated, simple and rapid bio-molecular assay to evaluate soluble HTT levels in blood cells as useful tool in disease and pharmacodynamic marker identification for observational and clinical trials.

## Background

Huntington’s disease is an inherited autosomal dominant neurodegenerative disorder characterized by motor dysfunction, psychiatric disturbances, and progressive dementia [1,2]. HD is caused by an unstable CAG repeat expansion in the gene encoding huntingtin (*HTT*) on chromosome 4, leading to an extended polyglutamine (polyQ) stretch in the amino terminus of the HTT protein [3]; the disease is therefore associated with a mutant form of the HTT protein that contains 36 or more glutamine residues. The presence of pathologic expanded HD alleles is detected by diagnostic testing in compliance with the Standards and Guideline for Clinical Genetics Laboratories; other scalable-throughput screening assay by PCR-MCA [4] or chimeric primed PCR (TP-PCR) [5] are developed and represent an attractive alternative to classical molecular screening method.

Pathogenesis arises mainly from mHTT expression, which leads to the formation of toxic soluble protein oligomers and insoluble aggregates [6,7], contributing to the disruption of multiple intracellular pathways involving mitochondrial dysfunction [8], oxidative stress [9], transcriptional dysregulation [10], autophagy [11,12] and metabolic impairment [13]. Nevertheless, loss of wild-type HTT function may also have a role in HD [14-16].

Several efforts are made to correlate the dysregulation of these pathways [17-19] with HD, providing solid platforms to describe disease progression. The pathology onset and severity significantly correlate with polyQ length [20], although environmental modulators and associated gene-environment interactions also influence disease progression [21,22]. Moreover HD is characterized by general brain atrophy and neuronal cell loss [23], which starts from the striatum and cortex, extending to other subcortical brain regions [24]. Whilst mHTT expression in the CNS [25] is the primary pathological hallmark in HD development [26], the presence of abnormalities in several other compartments [27] provide a source of accessible tissue for HTT quantification potentially to monitor disease progression and treatment efficacy. Here, we report the development of a robust and simple ELISA assay that is sensitive enough to detect differences in endogenous HTT levels in blood from HD patients at different stages of disease, highlighting its potential suitability for monitoring both disease progression and therapeutic intervention in clinical trials.

## Results

### Protein purification and quality control (QC)

**Figure 1:**
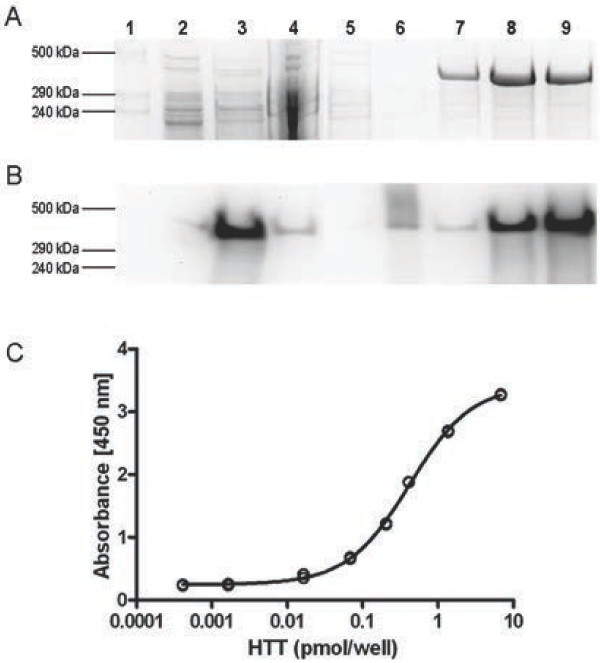
**Full-length HTT-Q138 purification and standard curve for HTT-ELISA assay.** Panel **A**, Gel coomassie staining of samples from the HTT-Q138 purification process (lane 1: molecular weight standard; lane 2: 30 μg RL1 cell lysate before induction; lane 3: 30 μg RL1 cell lysate 24 h post induction; lane 4: anti-FLAG affinity gel flow trough; lane 5–6: gel washes; lane 7–9: 5 μL of eluted fractions). Panel **B**, Western blot stained with anti-FLAG (lanes sequence as in panel **A**). Panel **C**, HTT-ELISA assay standard curve displays absorbance calibration values in duplicates individually. Curve showed is a representative example of multiple assays.

Recombinant human HTT full-length protein carrying a 3XFLAG tag at the N-terminus and a polyQ stretch of 138 glutamine residues (HTT-Q138) was produced using an inducible cell clone 293/T-Rex® Q138-CRE-RL1 (RL1) expressing intracellular HTT protein upon doxycycline induction [28]. To maximize yield and avoid significant degradation of the protein, induction times ranging from 12 to 96 hours were tested on small scale samples. We chose an induction time of 24 hours for the HTT-Q138 large scale preparation, since at that time HTT expression was stable. Figure 1A shows a typical result of the protein purification process. Purified HTT-Q138 showed an apparent molecular weight of ~340 kDa in agreement with the calculated value of 348 kDa. Recombinant protein was recognized by anti-FLAG antibody in cell lysate and efficiently captured by the same antibody immobilized onto the resin. Subsequently, it was eluted from the resin, by the competing FLAG-peptide; typically, we were able to obtain ~300 μg of HTT-Q138 from 1.2×10^9^ cells with a purity of more than 90%, as evaluated by Coomassie stained NuPAGE gels. The identity of the purified protein was confirmed by Western blotting using anti-HTT specific antibodies (data not shown). Tandem mass spectrometry analysis of purified protein samples, digested with three different enzymes (trypsin, Glu-C and chymotrypsin), identified 1044 unique peptides of the protein, which corresponded to a sequence coverage of 86% and confirmed the purity of HTT-Q138.

### Selection of antibodies for the HTT-ELISA

Several commercially-available antibodies (Table 1), raised against epitopes that were not overlapping with the polyQ region, were selected on the basis of their declared properties and literature description, with the aim of developing an ELISA sandwich assay able to quantify HTT protein in biological matrices (i.e. cell lysates or body fluids) irrespective of its polyQ expansion. The performance of each antibody as a capturer was assessed using purified HTT-Q138 as the standard protein and anti-FLAG-HRP conjugate as the detection antibody. Signal to background reading ratios were evaluated comparing four dilutions of each capture antibody against the standard curve, composed of concentrations ranging up to 5000 pg/well. The 4E10 and 3E10 antibodies were the most efficient, detecting HTT quantities up to 50 ng/well, reaching 18-fold signal-to-background ratio at saturation. The same procedure was then applied to select the best detection antibody. The most suitable was EP867Y and this was chosen together with 4E10 as the capture antibody to form the final HTT-ELISA. Subsequently, the assay conditions were optimized in terms of the concentrations of primary, secondary and HRP-conjugated antibody, incubation times and blocking agent to determine the maximally sensitive and stable assay conditions. These were 4E10 at 1 μg/mL, EP867Y at 1 μg/mL and blocking with BSA 1%. Under these conditions, the assay showed a dynamic range of five orders of magnitude (from 0.15 pM up to 30 nM of HTT), with a 19-fold signal-to-background ratio. Ten serial dilutions of HTT-Q138were used to generate standard curves in all subsequent analyses. Assay validation has been performed using ten independent experiments, obtaining intra-plate %CV below 10%, inter-assay %CV lower than 20%, LLOQ (lower limit of quantitation) of 2.7 fmol/well and accuracy within a 10% error. In each case, the standard curve was fitted with four-parameter sigmoid model and threshold for R square above 0.99 was set as acceptance criterion. An example of standard curve is presented in Figure 1C.

**Table 1:** Commercial antibodies tested

**Antibodies tested**		
**Name**	**Host**	**Antigen**
3D6	Mouse, monoclonal	aa 81-191
EP867Y	Rabbit, monoclonal	aa 511-588
D549	Rat, monoclonal	aa 549-679
3E10	Mouse, monoclonal	aa 997-1276
4E10	Mouse, monoclonal	aa 1844-2131
8A4	Mouse, monoclonal	aa 2703-2911

### Mutant and wild type HTT quantification in mammalian cell extracts

**Figure 2:**
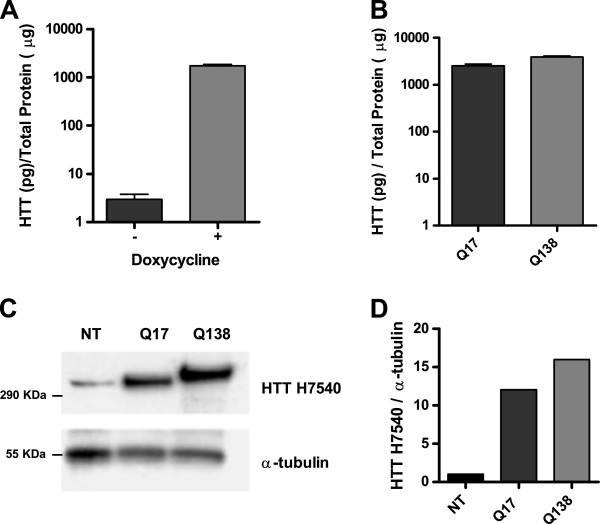
**HTT-Q138 and HTT-Q17 quantification in mammalian cell lysates sample.** Panel **A**, Fold-induction of HTT-Q138 expression detected with ELISA assay in uninduced and induced RL1 cells. Panel **B**, HTT-ELISA analysis of HEK293 cells transfected with full-length HTT-Q17 and HTT-Q138. Panel **C** and **D**, Western blot and densitometry analysis of HTT-Q17 and HTT-Q138 transfected HEK293 cells. The blot was probed with antibodies specific for HTT (HTT-H7540) and α-tubulin (loading control).

Total lysates of induced and non-induced RL1 cells, were used to establish the sensitivity and specificity of the HTT-ELISA on complex matrices. Since we were interested in quantifying only the soluble protein, a centrifugation step in lysates preparation was introduced to avoid any interference from HTT aggregates. This was adopted for all subsequent analyses. HTT-Q138 expression induced by 24 hours treatment with 1 μg/mL doxycycline was detected by our assay, showing an approximately 500-fold increase in HTT protein expression by these cells (Figure 2A). We also assessed the sensitivity of the assay for wild-type HTT relative to the mutant form, even though both molecular species should be detected with the same sensitivity (each of the selected antibodies recognize HTT epitopes far from the polyQ region, 4E10 at amino acids 1844–2131 and EP867Y at 511–588). We therefore verified the antibodies performance for the two proteins using total lysates of HEK 293 cells transiently transfected with plasmids encoding for 3XFLAG-full length HTT with either a stretch of 17 or 138 glutamine residues. 24 hours after transfection, cell lysates were analyzed by Western blotting with anti-HTT H7540 and by our HTT-ELISA assay (Figure 2B and C). The quantification of soluble HTT levels was in agreement with the densitometric quantification of Western blot analysis of the same samples (Figure 2D), demonstrating that the ELISA method was able to detect wild-type and mutant protein with the same sensitivity.

### Pharmacological assay validation

**Figure 3:**
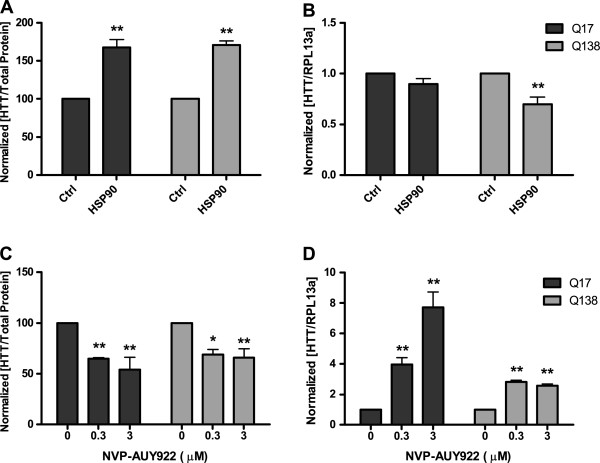
**Pharmacological assay validation.** Panel **A** and **B**, Effect of HSP90 overexpression on HTT protein (by ELISA) and gene (by RT-PCR) respectively. Panel **C** and **D**, Effect of HSP90 inhibition by NVP-AUY922 on HTT protein (by ELISA) and gene (by RT-PCR) respectively. Readings are normalized against Ctrl or DMSO as appropriate; statistical analyses are performed with two-way ANOVA followed by Bonferroni test for multiple comparisons (* p < 0.05, ** p < 0.01 wrt Ctrl or DMSO).

As inhibitors of HSP90 have been demonstrated to modulate mHTT steady state levels in cellular systems [29], we decided to validate our assay by assessing the detection of soluble HTT in complex matrices following pharmacological modulation. Firstly we verified that co-expression of HSP90 with wild-type and mutant HTT significantly increased the levels of HTT detected by the assay in total cell lysates (Figure 3A). This effect is exerted at protein level, as no increase in either HTT-Q138 or HTT-Q17 mRNA was observed by real-time qPCR and paradoxically, HTT-Q138 mRNA was reduced (Figure 3B). For pharmacological modulation, cells were treated for 24 hours with NVP-AUY922, a small molecule known to be a potent HSP90 inhibitor [30]. Upon modulation of HSP90 activity, we observed a significant reduction in soluble HTT protein irrespective of the presence of the expanded polyQ stretch (Figure 3C). Interestingly this treatment not only reduced the soluble protein, but also induced the expression of its mRNA as shown by RT-PCR analysis (Figure 3D). In summary, the pharmacological validation of the assay demonstrated its capacity to detect, in a significant manner, small variations in soluble HTT levels induced by inhibition of an enzyme modulating protein degradation.

### HTT detection in blood cells

**Figure 4:**
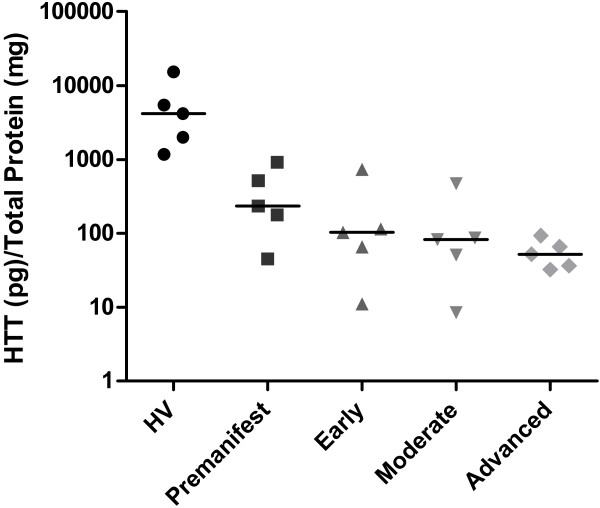
**HTT detection in blood cells sample.** Quantification by HTT-ELISA on human PBMC total lysates from HD patients at different disease stages, premanifest and healthy volunteers controls (HV). Gene carrier samples were significantly different from healthy controls (one-way ANOVA followed by Bonferroni test for multiple comparisons wrt HV group, p < 0.001). Individual estimates and median values are displayed in logarithmic scale.

A bioassay designed to measure soluble HTT level multiple times over long periods of time in clinical trials requires a minimally invasive procedure to obtain suitable material for analysis. Therefore, we chose PBMCs, as they are easily obtained from a patient’s blood sample. Preliminary experiments using lysates of fresh PBMCs obtained from 6 mL of pooled rat blood allowed us to measure HTT at a concentration of 0.25 ± 0.03 nM (mean ± SD). This demonstrated that the sensitivity of the assay was sufficient to quantify native protein using 100 μL/well of total lysate without using any sample enrichment procedure. To verify the suitability of the assay for the quantification of the HTT protein in specimens similar to those commonly available in clinical trials, we analyzed lysates from frozen human PBMC pellets. The complete set of samples, five subjects of each group, included healthy volunteers, premanifest and HD patients at early, moderate and advanced disease-stage. To ensure assay reproducibility, *in vitro* aggregation of high molecular weight HTT protein was prevented by processing each PBMC sample as described in the Methods section. Soluble HTT levels and total protein content were subsequently measured and results were expressed for each sample as ratio between HTT quantities and total protein content (Figure 4). HTT quantification was repeated in independent experiments, loading all the samples to be compared on the same plate to avoid inter-plate effects. The outcome of these series of experiments clearly demonstrated that HD patients have significantly lower levels of soluble HTT in their PBMCs compared with healthy controls. This negative modulation is even more evident when symptoms of the disease become manifest, suggesting altered HTT processing and clearance [31,32] as the disease progresses.

## Discussion

The site of the disease-causing mutation in the *HTT* gene responsible for HD was identified twenty years ago [33]. Since then, efforts have focused on the study of HTT and its role in pathogenesis, identifying the etiology of the disorder, treating and preventing motor symptoms, and managing a range of neurologic and behavioral complications [34-36]. The investigation has been challenging due to the high molecular weight of the HTT protein, its heterologous expression, and the tendency to aggregate [37-40]. Consequently, research has been directed at truncated forms [41] instead of the full-length protein. However, recent studies have started to examine the presence of the native full-length protein in human brain [42], leading to the generation of more physiological models of HD pathology [43,44] and suggesting that full length HTT may also be pathogenic in HD [45,46], thus boosting pharmaceutical research into drugs augmenting HTT clearance. The development of the assay is driven by the necessity to quantify in a precise and sensitive way the full length HTT protein in multiple biological matrices. During the development of the assay, we were able to identify suitable sandwich detection reagents from a wide selection of commercially available monoclonal antibodies against different epitopes of the full-length HTT protein. Importantly, the selected antibodies recognized not only the human HTT, but also the rodent homologue, facilitating quantification of the endogenous protein in animal models. Our ELISA has been demonstrated to be capable of detecting both the wild-type and mutant HTT protein with comparable sensitivity and to be very robust as the assay has been repeated over a period of more than two years, by different operators using several antibody lots giving always comparable results. The assay produced results in keeping with published data detecting a pharmacological modulation of HSP90 activity by means of its effect on soluble HTT levels in cultured cells. The analysis of human samples indicates that levels of soluble HTT in PBMC cells was quantifiable using our assay without any need of enrichment and that it was possible to detect different levels of the protein in healthy controls compared to HD patients. In fact, the decline in soluble HTT levels has already been shown to inversely correlate with disease-related aggregated HTT [47]. Interestingly, soluble HTT levels in premanifest mutation carriers are closer to those in HD patients with manifest disease than in healthy volunteers. We therefore speculate that the assay could be used as a valuable tool to monitor HTT concentrations longitudinally and to assess the efficacy of HTT lowering compounds in clinical trials and also in preclinical phase of the disease. Despite the interest of HTT quantification in peripheral tissues, only one assay, a TR-FRET for the detection of total and mutant HTT, has been published [48-50]. This homogeneous assay employs non-commercial antibodies and does not reveal differences in total HTT protein when comparing HD patients with healthy controls. The discrepancy of the results of the two assays could be explained in terms of different techniques, antibodies and analytes solubilization procedures used.

## Conclusions

The results presented here demonstrate that this HTT-ELISA is able to reliably detect the variation of HTT levels following pharmacological manipulation of an enzyme known to act on the steady-state levels of the protein. Further, it can differentiate between peripheral cells isolated from healthy controls and HD patients at different disease stages. This assay has recently been applied in a phase 1b clinical study performed at different sites, and represents a quick, easy and reliable tool to monitor the effects of potential therapeutics for HD in observational and clinical trials.

## Methods

### Recombinant human huntingtin expression and purification

The generation of recombinant 293/T-Rex® cells stably expressing, in a doxycyclin inducible manner, full-length mutant HTT with a stretch of 138 glutamines 3×Flag N-terminally tagged (T-Rex®-Q138-CRE-RL1), has been described elsewhere [28]. For large scale purification, 12×10^8^cells were grown in Dulbecco's Modified Eagle Medium, D-MEM containing 10% Tetra-Free Fetal Bovine Serum, 1% Pen/Strep, 1% G-Max (all from Gibco, Life Technologies, Paisley, UK) supplemented with 0.25 mg/mL Hygromycin (Life Technologies), 50 μg/mL Zeocin (InvivoGen, San Diego, CA) and 5 μg/mL Blasticidin (Invivogen) at 37°C, 5% CO_2_ in disposable 150 cm^2^ polystyrene flasks_._ Transgene expression was induced with doxycyclin (Sigma Aldrich, St. Louis, MO, USA) at 1 μg/mL final concentration and cells were collected after 24 hours. Cellular pellets were washed in PBS and lysed (10×10^6^ cells/mL) by sonication in loading buffer (Tris 50 mM pH 7.4, NaCl 150 mM, EDTA 1 mM) supplemented with protease and phosphatase inhibitors (Complete EDTA–free protease inhibitor cocktail, and PhosSTOP, phosphatase inhibitor cocktail, Roche Diagnostic GmbH, Mannheim, Germany). Total lysates were clarified by centrifugation at 1500 *g* for 5 minutes at 4°C. Typically, 10 mL of clarified lysates were loaded, in batch mode, onto 1 mL of slurry anti-FLAG M2 affinity gel (Sigma Aldrich) equilibrated in loading buffer, under moderate agitation, overnight at 4°C. Affinity gel was then washed twice with washing buffer (Tris 20 mM pH 7.4, NaCl 100 mM containing protease inhibitor) and HTT-Q138 protein was eluted in three 500 μL fractions of loading buffer containing 150 ng/μL 3×FLAG peptide for 30 minutes at 4°C. Each fraction was analyzed by Nu-PAGE on 3-8% Tris-Acetate gel (Life Technologies) followed by Bio Safe Coomassie Blue G-250 (Bio-Rad Hercules, CA, USA) staining and HTT identity was confirmed by Western Blotting using anti N-terminal HTT H7540 and anti FLAG antibodies. Protein concentration in eluted fraction was determined using BCA kit (Piercenet, Thermo Scientific, Rockford, USA) according to the manufacturer’s instructions.

### Western blot analysis

Total protein lysates (35 μg) were loaded on 3–8% Tris-acetate gels (Life Technologies) and transferred overnight at 30 V to PVDF membranes (GE Healthcare, Europe GmbH). Membranes were blocked in 3% NonFat Dry Milk (NFDM) for 1 hour, washed with PBS-Tween 20 0.01% and incubated with the appropriate antibody in 3% NFDM (anti-FLAG at 1 μg/mL, anti-N-terminal HTT H7540 at 0.1 μg/mL and anti α-tubulin, clone DM1A, all from Sigma Aldrich). HRP-conjugated secondary antibodies (Bio-Rad) were diluted 1:30,000 in 3% NFDM and incubated for 1 hour at room temperature. ECL Prime substrate (GE Healthcare) was used to develop chemiluminescent signal, acquired using Versadoc 4000 (Bio-Rad) or Hyperfilm ECL (GE Healthcare).

### Transient transfection and treatments

HEK293 cells were grown in D-MEM containing 10% FBS, 1% Pen/Strep, 1% G-Max (all from Gibco, Life Technologies). 8×10^5^ cells were seeded on MW6 plates (Corning Life sciences Inc.) coated with 1% poli-D lysine and after 24 hours transfected with 2 μg of total plasmid DNA using Lipofectamine 2000 (Life Technologies) according to manufacturer’s instructions. Plasmids carried the same HTT sequence used for stable cell line generation, bearing a stretch of 17 or 138 glutamines and an N-terminal 3XFLAG, under the control of CMV promoter (HTT-Q17 and HTT-Q138, respectively). Control wells were transfected with empty vector pcDNA 3.1 Zeo^+^ (Life Technologies). Medium was changed 4 hours after transfection and NVP-AUY922 (Novartis Institutes for Biomedical Research, Basel, Switzerland) in DMSO was added with medium change at final concentration of 0.3 and 3 μM. Control wells were supplemented with DMSO at the same concentration as test wells. Cells were collected 24 hours post-transfection and centrifuged. Pellets were resuspended in protein lysis buffer or RNA extraction buffer.

### Cell lysates preparation

Frozen pellets of PBMC from HD mutation carriers at premanifest, early, moderate and advanced stages and from healthy volunteers were analyzed. PBMC were isolated from whole blood collected in Mononuclear Cell Preparation Tubes (CPT with sodium citrate, DB Diagnostics) followed by density gradient centrifugation (1500 *g*, 20 minutes at room temperature). The PBMC layer was removed and washed twice with PBS (300 *g*, 10 minutes at room temperature). Cell pellets were snap-frozen and stored at -80°C until further analysis. Five cellular pellets from different subjects for each disease condition, (containing ~1.0 10^6^ cells, with vitality higher than 80%) were lysed by sonication in 1 mL physiological buffer (Tris 50 mM pH 7.4, NaCl 150 mM, EDTA 1 mM) with protease inhibitors (Complete EDTA–free protease inhibitor cocktail, Roche and PhosSTOP, phosphatase inhibitor cocktail, Roche). Total lysates were clarified by centrifugation at 3000 *g* for 5 minutes and their protein amount quantified by BCA (Piercenet) according to manifacturer’s instructions. Clarified samples were divided into single-use aliquots and stored frozen at -80°C. The same protocol was used to produce lysates of PBMC from rat blood and total lysates from RL1 clone and transfected HEK293 cells.

### RNA isolation and RT-PCR

Cells were collected 24 hours post transfection and RNA was isolated using RLT Buffer (RNeasy Plus Mini Kit, QIAGEN GmbH, Hilden, Germany) according to manufacturer’s instructions. 1 μg of mRNA was retrotranscribed using the QuantiTect Reverse Transcription Kit (QIAGEN) according to the manufacturer’s instructions. For every RNA sample two independent reverse transcriptase reactions were performed. Quantitative real-time RT-PCR (RT-qPCR) was performed in triplicate for the analyzed genes using the CFX96 Real Time System/C1000™ Thermal Cycler (Bio-Rad). All reactions were performed in a total volume of 20 μL containing 10 ng cDNA, 10 μL iQ™ SYBR Green Supermix (Bio-Rad) and 0.3 mM forward and reverse primers. Amplification cycles consisted of a first denaturating step at 95°C for 3 minutes, followed by 40 cycles of 30 seconds at 95°C and 30 seconds at 60°C. The amount of target gene mRNA was normalized to RPL13a levels. Primer sequences used were as follows: hsRPL13a_FWD CCTGGAGGAGAAGAGGAAAGAGA, hsRPL13a_REV TTGAGGACCTCTGTGTATTTGTC, hsHTT_FWD AAGCTCCCCCACCATTCG, hsHTT_REV TCTTGAGTGCTGGCAGATGCT.

### Enzyme-linked immunosorbent assay (ELISA)

ELISA assay for HTT quantification was performed on Nunc MaxiSorp 96-well ELISA plates (Thermo Scientific). The plates were coated overnight at 4°C with 100 μL/well of monoclonal mouse anti-HTT (clone HDB4E10, AbD Serotec, Bio-Rad lab, Inc.) antibody freshly diluted at 1 μg/mL in PBS. Plates were then washed three times in PBS with 0.1% Tween 20 (ELISA-washing buffer) and blocked with 300 μL/well of BSA 1% in PBS (ELISA-blocking buffer) for 30 minutes at room temperature. Standard HTT-Q138 protein and analytes were diluted in blocking buffer (100 μL final volume), added to the wells and incubated for 90 minutes at room temperature. After three washing steps, rabbit anti-HTT (clone EP867Y, Abcam plc, Cambridge, UK) was used as detection antibody diluted at 1 μg/mL in blocking buffer (100 μL/well) and incubated at room temperature for 60 minutes. After washing steps the immunocomplex was detected using an anti-rabbit IgG (H + L) HRP conjugated (Bio-Rad) antibody diluted 1:5000 in blocking buffer and incubated at room temperature for 60 minutes. After six washing steps 100 μL/well of TMB substrate (Sigma Aldrich) were added and colorimetric reaction was stopped after 10 minutes adding 100 μL/well of stop solution (Sigma Aldrich). The absorbance signals were read at 450 nm in Safire^2^ plate reader (Tecan Trading AG, Switzerland). A similar procedure was applied during assay development using different capture/detection antibodies and in optimization experiments. In cell lysates analysis, soluble HTT content in unknown samples, loaded in triplicate, was calculated through a standard curve (four parameter sigmoid model - IDBS XLfit4^™^) built by ten serial dilutions of HTT-Q138 from 1x10^-2^ to 2x10^3^ ng per well and loaded on the plate in duplicate. Standard calibrators having technical replicates with %CV higher than 10% were discarded from the fitting.

### Mass spectrometry and protein database search

HTT-Q138 purity was confirmed by MS/MS analysis performed at Proteome Sciences plc (United Kingdom). Protein identity was confirmed by MS/MS analysis. The purified HTT-Q138, separated by mono-dimensional SDS-PAGE, was in-gel digested using trypsin, endoproteinase Glu-C and chymotrypsin. Digests were analyzed using an electrospray (ESI) LTQ-Orbitrap mass spectrometer (Thermo Fisher Scientific) after reversed-phase nano liquid chromatography separation. A data-dependant acquisition method was used on top twenty ions selected after an MS survey scan. Selected ions were analyzed by MS/MS in the LTQ using collision-induced dissociation. Collected data were converted into peak lists and searched against protein databases using Mascot (Matrix Science Science Inc. MA, USA) through the Proteome Discoverer (Thermo Fisher Scientific) interface. Searches were performed in SwissProt and HTT-Q138 protein database built according to the Vector NTI file sequence carrying the 3XFLAG at the N-terminus of the protein.

### Statistical analysis

Pharmacological validation experiments data (HTT-ELISA and qPCR), were normalized on control sample in each transfection condition independently and statistical analysis was applied on normalized values by using a two-way ANOVA model considering transfection and treatment as independent variables. For qPCR data, statistical analysis was performed on logarithmic transformed HTT relative expression values additionally normalized on control samples.

HTT quantities measured in human PBMC from HD patients matched with healthy volunteers were analyzed using a one-way ANOVA considering, for each subject, average values obtained from the three technical replicates normalized by the total protein content. Where appropriate, post-hoc comparisons were performed with Bonferroni test.

## Authors’ contributions

LM, LP, LR and SB carried out all the experiments presented in the manuscript. LeM participated in the design of the study and performed the statistical analysis. SB, GP, AC and LM conceived the study and participated in its design and coordination. GP, LM, LP, SB, SH, RA and SDS wrote the manuscript. SDS, GBL, SH, RA and SJT provided PMBC samples from HD patients. All authors have carefully read and approved the final manuscript.
